# SNP55, a new functional polymorphism of *MDM2*-P2 promoter, contributes to allele-specific expression of *MDM2* in endometrial cancers

**DOI:** 10.1186/s12881-015-0216-8

**Published:** 2015-08-21

**Authors:** Kanako Okamoto, Ryosuke Tsunematsu, Tomoko Tahira, Kenzo Sonoda, Kazuo Asanoma, Hiroshi Yagi, Tomoko Yoneda, Kenshi Hayashi, Norio Wake, Kiyoko Kato

**Affiliations:** 1 Department of Obstetrics and Gynecology, Graduate School of Medical Sciences, Kyushu University, 3-1-1 Maidashi, Higashi-ku, Fukuoka, 812-8582 Japan; 2 Research Center for Environmental Medical Sciences, Kyushu University, Fukuoka, Japan; 3 Division of Genome Analysis, Research Center for Genetic Information, Medical Institute of Bioregulation, Kyushu University, Fukuoka, Japan; 4 Innovation Center for Medical Redox Navigation, Kyushu University, Fukuoka, Japan; 5 Current address: Department of Obstetrics and Gynecology, National Hospital Organization Ibusuki Medical Center, 4145, Junicho, Ibusuki, Kagoshima 891-0498 Japan

## Abstract

**Background:**

The functional single nucleotide polymorphism (SNP) in the *MDM2* promoter region, SNP309, is known to be associated with various diseases, particularly cancer. Although many studies have been performed to demonstrate the mechanism of allele-specific expression (ASE) on SNP309, they have only utilized *in vitro* techniques. It is unknown whether ASE of *MDM2* is ascribed solely to SNP309, *in vivo*.

**Methods:**

We attempted to evaluate ASE of *MDM2 in vivo* using post-labeling followed by automated capillary electrophoresis under single-strand conformation polymorphism conditions. For measuring a quantitative difference, we utilized the SNPs on the exons of *MDM2* as markers, the status of which was heterozygous in a large population. To address the cause of ASE beyond 20 %, we confirmed sequences of both *MDM2-*3’UTR and promoter regions. We assessed the SNP which might be the cause of ASE using biomolecular interaction analysis and luciferase assay.

**Results:**

ASE beyond 20 % was detected in endometrial cancers, but not in cancer-free endometria samples only when an SNP rs1690916 was used as a marker. We suspected that this ASE in endometrial cancer was caused by the sequence heterogeneity in the *MDM2*-P2 promoter, and found a new functional polymorphism, which we labelled SNP55. There was no difference between cancer-free endometria and endometrial cancer samples neither for SNP55 genotype frequencies nor allele frequencies, and so, SNP55 alone does not affect endometrial cancer risk. The SNP55 status affected the DNA binding affinity of transcription factor Sp1 and nuclear factor kappa-B (NFκB). Transcriptional activity of the P2 promoter containing SNP55C was suppressed by NFκB p50 homodimers, but that of SNP55T was not. Only ASE-positive endometrial cancer samples displayed nuclear localization of NFκB p50.

**Conclusions:**

Our findings suggest that both the SNP55 status and the NFκB p50 activity are important in the transcriptional regulation of *MDM2* in endometrial cancers.

## Background

Normal mammalian somatic cells have a limited lifespan. Cells are able to divide only a limited number of times, and then undergo senescence. If the cellular senescence system is impaired by an insult such as an epigenetic change or a somatic mutation normal cells acquire the ability to proliferate for an unlimited period (immortalization). This is an essential step that initiates tumorigenesis in the malignant transformation of normal cells [1–6]. Inheritable genome variations were also recently shown to disrupt cellular senescence system [7–9]. Genome wide association studies (GWAS) have revealed an association between genetic variations and common diseases, and that most of these polymorphisms do not change the protein sequence [10, 11]. Some of the single nucleotide polymorphisms (SNPs) in gene promoter regions, termed rSNPs, have a potential to modulate gene expression.

If rSNP exists as a heterozygous state, a quantitative difference in gene expression between the two alleles should be observed. This phenomenon is called allele-specific expression (ASE) [12–20]. A previous study about ASE revealed that six out of 13 genes showed more than a 20 % difference in gene expression between the two alleles [12]. Lo et al. [17] showed that ASE was detected in 54 % of genes (326/602), which was investigated using Affymetrix® HuSNP oligo array in the kidneys and livers from seven individuals; they also identified that most imbalanced expression was associated with autosomal genes and a few imprint genes. Another study detected ASE in 53 % of genes (731/1389) in the leukocytes from 12 unrelated individuals, [19]. Many studies have confirmed the association between diseases, particularly cancer, and rSNPs in various autosomal genes [*APC* [9], *TGFBR1* [21], *AGTR1* [22], *FSHB* [23, 24], *BRCA1* and *BRCA2* [25], *CRAC1* [26], *CDH1* [27], *MDM2* [28–30]].

*Mdm2* was originally identified as an amplified proto-oncogene in the BALB/c cell line 3T3DM [31]. The major function of Mdm2 is a negative regulation of the p53 tumor suppressor protein. Mdm2 has E3 ubiquitin ligase activity; it binds to the transcriptional activation domain of p53 leading to proteasome-mediated degradation [32–35]. Cloning of the human homolog of Mdm2 allowed the confirmation of the interaction with p53, which indicated that *MDM2* contributes to tumorigenesis. *MDM2* [GenBank: AF527840] is amplified in 30–40 % of sarcomas [36, 37] as well as various cancers [38]. Several reports describe MDM2 overexpression in certain cancers without gene amplification, such as leukemia [39], melanoma [40, 41], and breast cancer [42, 43].

*MDM2* is transcribed from two promoters; the one that is upstream of exon 1 (the P1 promoter) and the other that is in intron 1 (the P2 promoter) [44–46]. The *MDM2*-P1 promoter works for basal transcription independently of p53, whereas the *MDM2*-P2 promoter is considered to be inducible by p53. There is a difference in the 5’ untranslated region (UTR) sequence between P1- and P2-derived mRNAs. ASE was reported in both *MDM2*-P1 and *MDM2*-P2 promoters. C1797G (rs937282; C/G) polymorphism in the *MDM2*-P1 promoter may affect transcription by altering the binding affinity of CCAAT-enhancer-binding protein (C/EBPα) to the promoter. It is also associated with bladder cancer risk [29]. In the *MDM2*-P2 promoter, SNP285 (rs117039649; C/G) [30, 47, 48], SNP309 (rs2279744; G/T) [28, 49], and SNP344 (rs1196333; A/T) [50] were reported, of which SNP309 is particularly well investigated. SNP309G enhances MDM2 expression from the P2 promoter by increasing the binding affinity of the transcription factor Sp1. Therefore, a large number of retrospective analyses were conducted showing an association between SNP309G and cancer risk [51–56]. However, other studies have failed to confirm this association [57–61], and the results remain inconsistent for endometrial cancer [51, 62–66].

In this study, we attempted to confirm that ASE of *MDM2* occurs *in vivo* in cancer-free endometria and in endometrial cancer. We identified a new functional polymorphism, SNP55 (rs2870820; C/T), which causes ASE of *MDM2*. SNP55 status alone had no association with endometrial cancer risk. We revealed that SNP55C suppressed the transcriptional activity of the *MDM2*-P2 promoter by recruiting the nuclear factor kappa-B (NFκB) p50 homodimer.

## Methods

### Tissue samples and cell culture

The use of human tissue in this investigation was reviewed and approved by the ethical committee of Kyushu University. Cancer-free endometrial tissues (*n* = 45) were collected from hysterectomy specimens removed for the treatment of uterine myomas. Endometrial cancer samples (*n* = 45) were obtained from women who underwent surgery. In all case, written consent for tissue donation was obtained from participants. COS-1 cells were maintained in Dulbecco’s modified Eagle’s medium (DMEM) (Sigma) supplemented with 10 % calf serum (Nichirei Biosciences) and 1 % penicillin/streptomycin (Gibco). Cells were cultured at 37 °C in a humidified 5 % CO_2_ atmosphere.

### Preparation of genomic DNA, RNA, and cDNA

Genomic DNA and RNA were concurrently extracted from endometrium specimens (both with cancer and cancer-free) using AllPrep DNA/RNA Mini Kit (Qiagen). An aliquot of the total RNA (500 ng) was reverse transcribed to cDNA using random primer and ReverTra Ace-α® (Takara).

### Genotyping

Both SNP55 and SNP309 in the *MDM2*-P2 promoter region were identified via PCR amplification followed by sequencing the amplified PCR fragments. PCR primers (Forward: 5′-CGAGCTTGGCTGCTTCTGGG-3′, Reverse: 5′-GCTGGAATCTGTGAGGTGGT-3′) were designed against the sequence around these two SNPs to amplify approximately 1 kbp of the product using KOD-FX (TOYOBO). PCR products were separated by a 1 % agarose gel and excised. They were purified using GEX^TM^ PCR DNA and Gel Band Purification Kit (GE Healthcare). Purified PCR products were sequenced using a sequencing primer (5′-AGCAAGTCGGTGCTTACCTG-3′). Sequencing reactions were conducted using BigDye Terminator v3.1 Cycle Sequencing Kits (Applied Biosystems). Two marker SNPs, rs1690916 (A/G) and rs937283 (A/G), were genotyped by post-labeling followed by automated capillary electrophoresis (PLACE) under single-strand conformation polymorphism (SSCP) conditions (the PLACE-SSCP method) using genomic DNA.

In order to determine the haplotype involving SNP55 and SNP309, we cloned the relevant region and sequenced. The *MDM2*-P2 promoter region of genomic DNA was amplified by PCR with KOD-FX (TOYOBO) using forward primer, 5′-TATCTCGAGGTACTGGCCCGGCAGCGA-3′ and reverse primer, 5′-TATAAGCTTGAACACAGCTGGGAAAATGC-3′. PCR products were inserted into *Eco*RV site of pBluescript® (Stratagene) and the insert regions were sequenced.

### PLACE-SSCP and *in vitro* transcription

DNA fragments around marker SNPs were amplified by PCR. For marker SNP rs1690916, PCR amplification was performed using Ampli Taq DNA polymerase (Applied Biosystems) and the following primers: forward, 5′-attATCAGGCCCTTTTTCACACA-3′ and reverse, 5′-gttACCCAGGCCAAGAAGGTACT-3′ (product size 122 bp). The “att” and “gtt” sequences were tags attached for the purpose of fluorescent post-labeling [67]. For marker SNP rs937283, PCR was performed using KOD-FX (TOYOBO) and the following primers: forward, 5′-attCTGGCCCGGAGAGTGGAAT-3′ and reverse, 5′-gttAATGGTCCCGTTTTCGCGCTTGGAGTC-3′ (product size 124 bp), and because the initial attempt revealed poor separation of the two alleles of SNP rs937283, we attached 9-bp extra-sequences (underlined) to the N-terminus of the reverse primer, so that only the PCR product from the G-allele of SNP rs937283 could form a stem-loop structure. The details of the PLACE-SSCP method have been described previously [67]. The analysis of marker SNP rs937283 was performed using 0.094 μl of Thermo Sequenase (GE Healthcare) per sample for post-labeling, which was ten times greater than usual, and SSCP electrophoresis was performed at 42 °C.

Precise quantification of the alleles in cDNA was performed by synthesizing four RNA fragments corresponding to the two marker SNPs (A/G for rs937283 and G/A for rs1690916) by *in vitro* transcription. Templates were produced by PCR amplification using the same primers as those used for PLACE-SSCP. Templates were subcloned into pBluescript® (Stratagene) and subjected to *in vitro* transcription using an in vitro transcription T7 kit (Takara). Template DNA was digested with DNase (Promega). Following phenol and chloroform extractions, the produced RNA was purified and concentration was measured by NanoDrop™ (Thermo Scientific).

### Transcription factor Sp1 and NFκB p50-DNA binding analysis

Biomolecular interaction analysis (BIA) was performed on the Biacore3000 (GE Healthcare). Sensor chip SA was used to anchor one of the two different double-stranded DNA 22-mers (5′-GACGGTGTCCC/TTTCTATCGCTG-3′) representing the *MDM2*-P2 promoter with different SNP55 status, of which the N-terminus was biotinylated. To confirm that only double-stranded DNA fragments were immobilized, single-stranded binding proteins were injected at a concentration of 2.5 μg/ml with a flow 5 μl/min for 1 min and no signals were detected (data not shown). At a level of 2000 response units (RU), DNA fragments were immobilized in each flow cell. Transcription factor Sp1 (Proteinone), extracted from HeLa cells, or recombinant NFκB p50 (Enzo) was prepared at a concentration of 1 μg/ml, in advance. Both proteins were injected in flow cells and a reference surface (blank) for comparison with a 5 μl/min flow for 5 min at 25 °C. Biosensor data were analyzed using the BIA evaluation software.

### Construction of plasmids

The *MDM2*-P2 promoter region of genomic DNA was amplified by PCR. PCR products containing either SNP55T or SNP55C were inserted in tandem into a pGL4 luciferase reporter plasmid (Promega). PCR of the *MDM2*-P2 promoter region was performed using the following primers: forward, 5′-TATCTCGAGGTACTGGCCCGGCAGCGA-3′ and reverse, 5′-TATAAGCTTGAACACAGCTGGGAAAATGC-3′, including the *Xho*I and *Hind*III restriction site. We confirmed that the clones had different SNP55 statuses only, and that the other sequences were identical. We constructed expression vectors, pcDNA-myc-Sp1 and pcDNA-FLAG-NFκB p50, as follows. Transcription factor Sp1 and NFκB p50 were amplified from the cDNA of MCF-7 cell lines by KOD-FX (TOYOBO). During PCR, we attached a myc-tag to the N-terminus of transcription factor Sp1 using the following primers: forward, 5′-TATGAATTCGCCACCATGGAACAGAAACTGATCTCTGAAGAAGACCTGAGCGACCAAGATCACTCC-3′ and reverse, 5′-ATACTCGAGgTCAGAAGCCATTGCCACTGAT-3′ and the FLAG tag to the N-terminus of NFκB p50 using the following primers: forward, 5′-TATGAATTCGCCACCATGGACTACAAAAGACGATGACGACAAGGCAGAAGATGATCCATAT-3′ and reverse, 5′-ATACTCGAGCTAACTTCCAGTGCCCCCTCC-3′, including the *Eco*RI and *Xho*I restriction site. The amplified fragments and pcDNA3 (Invitrogen) were ligated using the *Eco*RI and *Xho*I enzymes (Takara Biotech Co) and were subcloned into pcDNA3.

### Transfection and luciferase assay

COS-1 cells (2 × 10^5^ cells/6 wells) were transfected with 1 μg of pGL4 reporter plasmids using Lipofectamine 2000 (Invitrogen) according to the manufacturer’s instructions. Reporter plasmids and expression vectors were co-transfected (Fig. 6). Cells were harvested 24 h after transfection. Luciferase activity was determined using the Luciferase Assay System (Promega). For correction of variations, the cultured cells were co-transfected with a β-galactosidase expression vector, pcDNA-LacZ. Luciferase activity was normalized according to the β-galactosidase activity. Each transfection was performed in triplicate.

### Western blot analysis

Cell extracts were prepared using an ice-cold lysis buffer (50 mM Tris–HCl [pH7.6], 300 mM NaCl, 0.5 % Triton-X, 400 μM Na_3_VO_4_, 400 μM EDTA, 10 mM NaF, 10 mM Na pyrophosphate, 10 μg/ml Aprotinin, 10 μg/ml Leupeptin, 10 mM lodoacetamide, and 1 mM PMSF) for 15 min. Protein concentrations were measured by Protein Assay (Bio-rad). An equal amount of cell lysate was separated by sodium dodecyl sulfate polyacrylamide gel electrophoresis (SDS-PAGE) (10 % gel). Proteins were then transferred to polyvinylidene difluoride membranes (GE Healthcare). To avoid nonspecific binding, the membranes were incubated for 1 h at room temperature with tris-buffered saline (TBS) containing 0.05 % Tween-20 and 5 % nonfat dry milk, before being probed by respective antibodies. The primary antibodies used were transcription factor Sp1, NFκB p50, and glyceraldehyde 3-phosphate dehydrogenase (GAPDH; Santa Cruz Biotechnology). After washing in TBS containing 0.05 % Tween-20, the membranes were incubated with the secondary antibodies, anti-mouse or anti-rabbit horseradish peroxidase (HRP) (Promega). Chemiluminescence was visualized using the Clarity Western ECL substrate (Bio-rad).

### Immunohistochemistry

Immunohistochemical staining of NFκB p65 and p50 were performed on four endometrial cancer sections (two sections were ASE-positive and the other two were ASE-negative) as follows. For pretreatment, sections were blocked by the Protein block (Dako) for 10 min at room temperature and incubated overnight at 4 °C with the primary antibodies, p65 (1:100) and p50 (1:100) (Santa Cruz Biotechnology) diluted in antibody diluent (Dako). Endogenous peroxidase was inactivated using 3 % hydrogen peroxidase (Sigma). The primary antibody in control experiments was replaced with normal goat IgG (Santa Cruz Biotechnology). After incubation with Envision™ + Rabbit/HRP (Dako) for 30 min, signals were developed using 3.3-diaminobenzidine (DAB). The sections were then counterstained with hematoxylin and mounted.

### Statistical analysis

Differences between two groups were evaluated using the Student *t* test. The genotype frequencies and allele frequencies were compared using Fisher’s exact test. The *P*-values given were two-sided and a *P*-value of ‹0.05 was considered statistically significant.

## Results

### Selection of marker SNPs and optimization of PLACE-SSCP

**Fig. 1:**
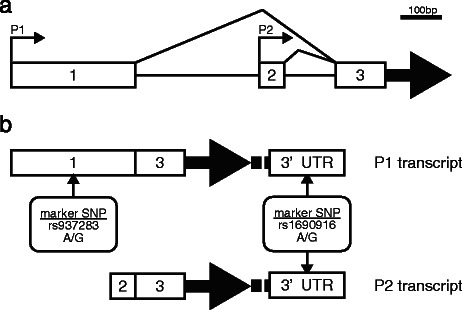
Genomic organization of the human *MDM2* gene. (**a**) The transcription start sites of the two promoters are indicated. (**b**) The two transcriptional products from the P1 and P2 promoters have different 5’ (untranslated region) UTR sequences. All *MDM2* RNAs have the same 3’ UTR sequences, in which single nucleotide polymorphism (SNP) rs1690916 is located. SNP rs937283 is on exon 1, therefore only transcripts from the P1 promoter include this SNP

**Fig. 2:**
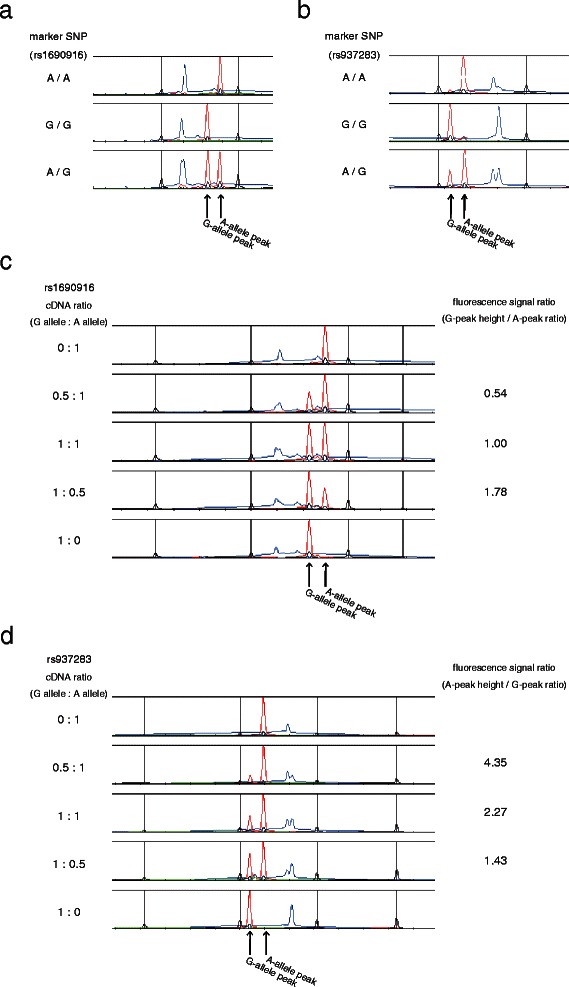
Application of PLACE-SSCP methods for the evaluation of ASE. **a**, **d** Genotyping marker single nucleotide polymorphism (SNP) rs1690916 (**a**) and rs937283 (**b**) by genomic DNA from cancer-free endometria. **c**, **d** Confirmation of precise measurement by PLACE-SSCP with marker SNP rs1690916 (**c**) and rs937283 (**d**) cDNA. RNA was transcribed *in vitro* and reverse-transcribed. The mixture rate between each allele is shown on the left

The SSCP method is sensitive for detecting single base sequence differences in PCR-amplified DNA fragments as separated peaks in electrophoretic analysis, whereas the subsequent development of PLACE-SSCP is suitable for precise estimation of SNP-allele frequency when applied to pooled DNA [67]. Here, we attempted to utilize the PLACE-SSCP method for evaluating ASE. To distinguish mRNAs produced from two alleles and measure a quantitative difference, we utilized the SNPs on the exons of *MDM2* as markers, the status of which was heterozygous in a large population. Because SNP rs1690916 is located at the 3’UTR of *MDM2*, it was used to detect ASE in the *MDM2* total mRNA. We selected SNP rs937283, located at exon 1 of *MDM2*, as another candidate marker SNP. Using SNP rs937283 as a marker, we can measure only the quantitative differences in mRNAs produced from the *MDM2*-P1 promoter (Fig. 1a, b). At first, we confirmed whether each marker SNP status was detectable by genomic DNA (Fig. 2a, b). Initial experiments revealed poor separation of the two alleles of SNP rs937283 (data not shown). Clean separation, with two fluorescent signal peaks, was achieved by attaching a short complementary sequence to the G-allele marker of SNP rs937283 at the 5’ position.

We also noted that the fluorescence signal ratio of two alleles of SNP rs937283 was not close to 1.0 in cancer-free heterozygous samples. To precisely quantitate the expression of the two alleles in cDNA, via *in vitro* transcription we created two RNA fragment patterns in which only the marker SNP status was different. We then made an equal concentration mixture of these artificial RNAs and reverse-transcribed them to cDNA. PLACE-SSCP using these cDNA demonstrated equal expression levels of cDNA from heterozygote of cancer-free samples (Fig. 2c, d).

### ASE was detected in endometrial cancers, but not in cancer-free endometria

**Fig. 3:**
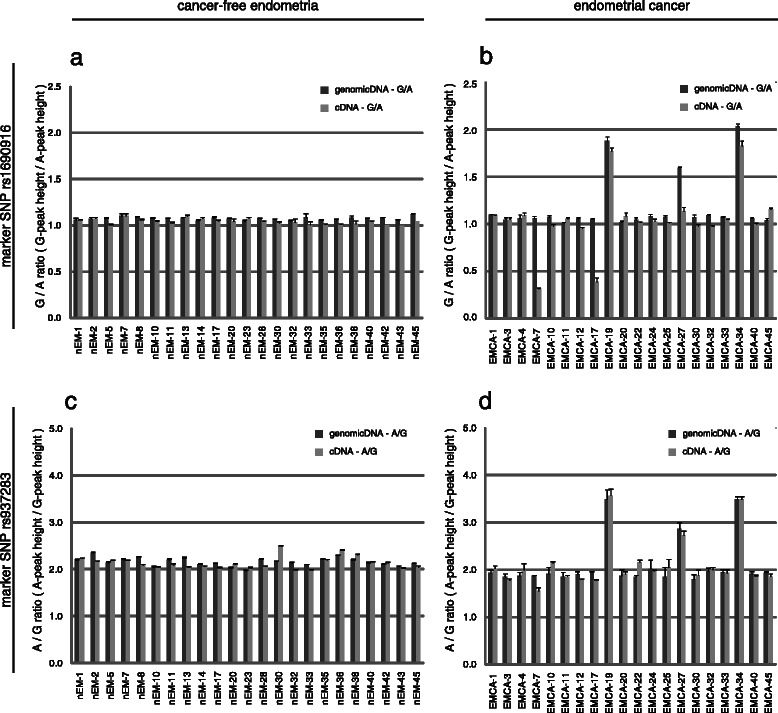
ASE of *MDM2* mRNA analyzed by PLACE-SSCP method. Allelic ratio in genomic DNA and relative allele-specific expression ratio in cDNA from the same samples that analyzed by the post-labeling followed by automated capillary electrophoresis under single-strand conformation polymorphism conditions (PLACE-SSCP) method with either marker single nucleotide polymorphism (SNP) rs1690916 (**a**), (**b**); or marker SNP rs937283 (**c**), (**d**); in cancer-free endometria (**a**), (**c**); and endometrial cancer (**b**), (**d**). The y-axis represents the fluorescent signal ratio from each allele (SNP rs1690916: G-peak height/A-peak height; SNP rs937283: A-peak height/A-peak height). Dark grey bars indicate the corrected genomic allelic ratios and light grey bars indicate the corrected cDNA allelic ratios

We genotyped the marker SNPs in cancer-free endometria (*n* = 45) and identified heterozygous SNP rs937283 (*n* = 23) and SNP rs1690916 (*n* = 23; Tables 1 and 2). Their genomic DNA and cDNA were analyzed by PLACE-SSCP. The fluorescence signal ratio of each allele (SNP rs1690916: G-peak height A-peak height, SNP rs937283: A-peak height/G-peak height) was used to evaluate ASE and we could accurately measure quantitative differences between the two alleles (Fig. 2c, d). At SNP rs1690916, the ratio in cDNA from all 23 measured cancer- free endometria was similar, and no significant difference (more than 20 %) was detected between allelic ratios in genomic DNA and cDNA of each sample (Fig. 3a). Yan and colleagues employed the threshold of the 20 % relative difference to evaluate ASE [12]. Next, we observed ASE using SNP rs937283, but ASE from the *MDM2*-P1 promoter was not identified (Fig. 3c).

**Table 1:** Genotype of marker SNP rs1690916 in samples of cancer-free endometria and endometrial cancer

3’UTR marker SNP rs1690916	Cancer-free endometria	Endometrial cancer (EMCA)
A/A	4	6
A/G	23	20
G/G	18	19
total	45	45

**Table 2:** Genotype of marker SNP rs937283 in samples of cancer-free endometria and endometrial cancer

Exon1 marker SNP rs937283	Cancer-free endometria	Endometrial cancer (EMCA)
A/A	18	19
A/G	23	20
G/G	4	6
total	45	45

We applied the same methods to endometrial cancer samples. Of the total 45 endometrial cancer patients, 20 showed heterozygous SNP rs1690916 and SNP rs937283 (Tables 1 and 2, respectively). Three endometrial cancer samples (EMCA-19, 27, 34) represented mono-allelic amplification; thus, they were excluded from the ASE evaluation. ASE beyond 20 % at marker SNP rs1690916 was observed in 2 of 17 heterozygous specimens (Fig. 3b). With regard to the cancer-free individuals, we attempted to examine ASE at SNP rs937283 but were unable to detect expression differences of more than 20 % in all samples that were heterozygous for this marker SNP (Fig. 3d).

### ASE in endometrial cancer is associated with a new functional SNP

ASE was not identified in endometrial cancer samples using marker SNP rs937283, suggesting that expression differences between alleles did not originate from the *MDM2*-P1 promoter. Thus, it was assumed that there are two possible mechanisms of generating quantitative differences from each allele, degradation of mRNA or different transcription levels (more/less) between alleles. To address the cause of ASE in endometrial cancer, we assessed these two hypotheses. We checked the former hypothesis by confirming the 3’ UTR sequences of two ASE-positive samples; however, we identified no genomic variations that affected the stability of transcribed mRNA, such as consensus sequences of microRNAs.

**Fig. 4:**
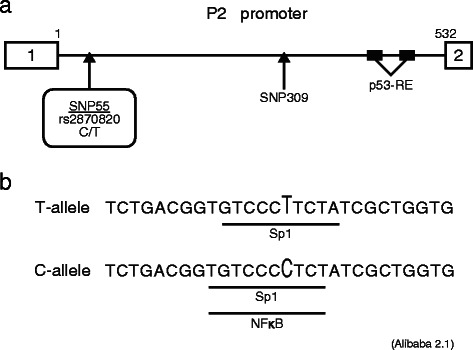
*MDM2*-P2 promoter contains a SNP which affects the binding affinity of transcription factors. **a** Schematic illustration of the *MDM2*-P2 promoter. Single nucleotide polymorphism (SNP) 55 is upstream of SNP309 and two p53 responsive elements. **b** SNP55 alters transcription factors that can bind to DNA around SNP55 (analyzed by Alibaba 2.1)

To examine the latter hypothesis, we checked *MDM2*-P2 promoter sequences. In two ASE-positive individuals with endometrial cancer, one had T/T and the other had T/G at SNP309. SNP55 (rs2870820) was also heterozygous (Fig. 4a).

We genotyped both SNP55 and SNP309 status in both cancer-free endometria (*n* = 45) and endometrial cancer samples (*n* = 45) analyzed by genome DNA sequencing (Table 3). Both genotype frequencies were found to be consistent with the Hardy-Weinberg’s equilibrium. There was no difference between cancer-free endometria and endometrial cancer samples neither for the genotype frequencies, nor allele frequencies (Table 4). We then investigated the haplotype frequency of the region involving SNP55 and SNP309 in cancer-free endometria. Frequency of each haplotype is as follows: (55C-309 T) = 0.178; (55C-309G) = 0.467; (55 T-309 T) = 0.356; (55 T-309G) = 0.000. Linkage disequilibrium (r^2^) between SNP55 and SNP309 was calculated as 0.483 from these haplotype frequencies.

**Table 3:** Histological classification and genotype of endometrial cancer (EMCA) samples

	Age	Histological classification (grade)	Exon 1 marker SNP (rs937283)	3’ UTR marker SNP (rs1690916)	SNP55 (rs2870820)	SNP309 (rs2279744)
EMCA-1	53	Endometrioid(G1) + Squamous cell	A/G	A/G	C/T	T/G
EMCA-2	80	Endometrioid(G3)	A	G	C	G
EMCA-3	50	Endometrioid(G2) + Squamous cell	A/G	A/G	C/T	T
EMCA-4	51	Serous	A/G	A/G	C/T	T
EMCA-5	71	Endometrioid(G1)	G	A	T	T
EMCA-6	74	Serous	G	G	C/T	T
EMCA-7	76	Mixed + Serous	A/G	A/G	C/T	T
EMCA-8	42	Endometrioid(G1)	A	G	C	T/G
EMCA-9	58	Endometrioid(G1)	A	G	C	G
EMCA-10	54	Endometrioid(G3)	A/G	A/G	C/T	T/G
EMCA-11	39	Endometrioid(G1)	A/G	A/G	C/T	T/G
EMCA-12	62	Endometrioid(G2)	A/G	A/G	C/T	T
EMCA-13	71	Hetero	G	A	T	T
EMCA-14	66	Endometrioid(G2)	A	G	C	G
EMCA-15	81	Serous	A	G	C	T/G
EMCA-16	37	Endometrioid(G1)	A	G	C	G
EMCA-17	70	Endometrioid(G1)	A/G	A/G	C/T	T/G
EMCA-18	43	Endometrioid(G1)	G	A	T	T
EMCA-19	61	Serous	A/G	A/G	C/T	T/G
EMCA-20	77	Endometrioid(G2)	A/G	A/G	C/T	T/G
EMCA-21	74	Endometrioid(G1)	A	G	C	G
EMCA-22	56	Endometrioid(G2)	A/G	A/G	C/T	T/G
EMCA-23	42	Endometrioid(G1)	A	G	C	G
EMCA-24	55	Endometrioid(G1)	A/G	A/G	C/T	T/G
EMCA-25	69	Endometrioid(G1)	A/G	A/G	C/T	T/G
EMCA-26	65	Endometrioid(G1)	A	G	C	T/G
EMCA-27	56	Endometrioid(G2)	A/G	A/G	C/T	T
EMCA-28	83	Endometrioid(G2)	A	G	C	G
EMCA-29	58	Endometrioid(G2)	A	G	C	T
EMCA-30	78	Serous	A/G	A/G	C/T	T/G
EMCA-31	74	Endometrioid(G1)	A	G	C	G
EMCA-32	35	Endometrioid(G1)	A/G	A/G	C/T	T/G
EMCA-33	57	Endometrioid(G1)	A/G	A/G	C/T	T/G
EMCA-34	52	Endometrioid(G3) + Clear cell	A/G	A/G	C/T	T/G
EMCA-35	55	Endometrioid(G2)	G	A	T	T
EMCA-36	49	Endometrioid(G2)	A	G	C	T/G
EMCA-37	60	Endometrioid(G1)	A	G	C	G
EMCA-38	56	Endometrioid(G3)	A	G	C/T	T/G
EMCA-39	60	Endometrioid(G1)	G	A	T	T
EMCA-40	64	Endometrioid(G1)	A/G	A/G	C/T	T/G
EMCA-41	60	Endometrioid(G2)	G	A	T	T
EMCA-42	48	Endometrioid(G1)	A	G	C	G
EMCA-43	58	Clear cell	A	G	C	T/G
EMCA-44	58	Clear cell	A	G	C	T/G
EMCA-45	55	Endometrioid(G1)	A/G	A/G	C/T	T

**Table 4:** Genotype and allele frequencies of *MDM2* SNP55 and SNP309 in cancer-free endometria and endometrial cancer samples

	Genotype				Alleles		
SNP55 rs2870820	C/C	C/T	T/T	*p*-value	C	T	*p*-value
Cancer-free endometrial (*n* = 45)	17 (37.8 %)	24 (53.3 %)	4 (8.9 %)		58 (64.4 %)	32 (35.6 %)	
Endometrial cancer (EMCA) (*n* =45)	17 (37.8 %)	22 (48.9 %)	6 (13.3 %)	0.87	56 (62.2 %)	34 (37.8 %)	0.88
SNP309 rs2279744	T/T	T/G	G/G	*p*-value	T	G	*p*-value
Cancer-free endometrial (*n* = 45)	12 (26.7 %)	24 (53.3 %)	9 (20.0 %)		48 (53.3 %)	42 (46.7 %)	
Endometrial cancer (EMCA) (*n* =45)	14 (31.1 %)	21 (46.7 %)	10 (22.2 %)	0.85	49 (54.4 %)	41 (45.5 %)	1.00

### The effect of SNP55 on the binding affinity of transcription factors to the *MDM2*-P2 promoter region

**Fig. 5:**
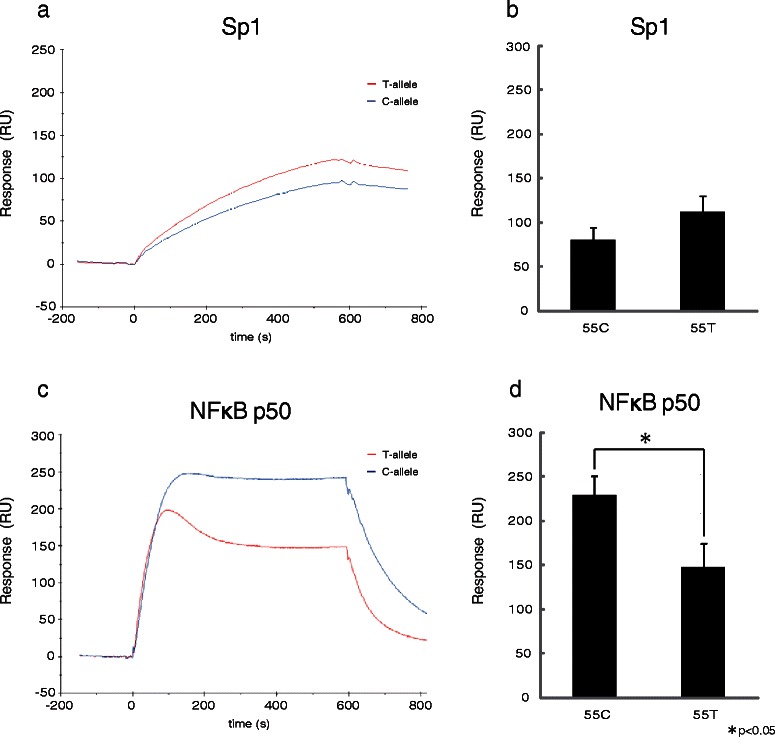
Biomolecular interaction analysis (BIA) using Biacore. **a** Biosensor figure of relative quantitative response with transcription factor Sp1, purified from HeLa cells. **b** Comparison of a response unit (RU) value at 600 s for (**a**). **c** Biosensor figure of the relative quantitative response with recombinant nuclear factor kappa-B (NFκB) p50. **d** Comparison of the RU value at 600 s for (**c**)

To investigate the effect of SNP55 on the *MDM2*-P2 promoter transcriptional activation, we utilized a computer algorithm (Alibaba 2.1) and found that both the SNP55T and SNP55C alleles could bind transcription factor Sp1, but that only SNP55C had consensus sequence to NFκB (Fig. 4b). This result indicated that the SNP55 status may affect the binding affinity of transcription factor Sp1 and NFκB. To confirm the hypothesis, we conducted BIA. We focused on the p50 transcription factor, from the NFκB/Rel family, because both transcription factor Sp1 and NFκB p50 recognized the same DNA binding site and functionally interfere with each other [68]. Transcription factor Sp1 bound, insignificantly, but more easily to SNP55T than SNP55C (Fig. 5a, b). On the other hand, NFκB p50 significantly generated more DNA–protein complexes with SNP55C (Fig. 5c, d; P ‹0.05). These results indicate that the SNP55 status may affect the transcription activity of the *MDM2*-P2 promoter.

### Only the SNP55C allele was suppressed by NFκB p50 in a dose-dependent manner

**Fig. 6:**
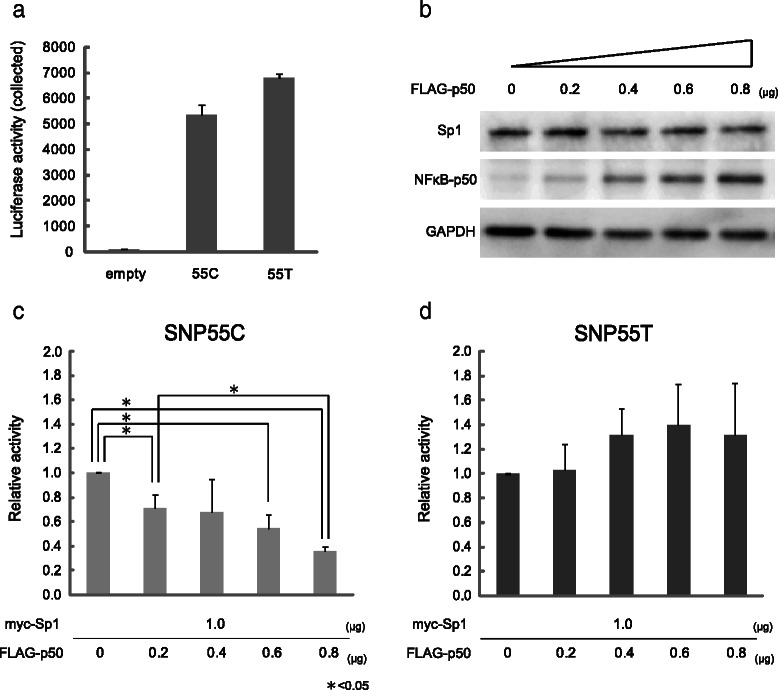
The effect of SNP55 on *MDM2*-P2 promoter activity. **a** The pGL4 luciferase reporter plasmids containing either SNP55T or SNP55C were co-transfected with pcDNA-LacZ (control) and pcDNA-Sp1 (N-terminus myc-tagged). **b** Western blot analysis confirming a gradient increase of nuclear factor kappa-B (NFκB) p50. Glyceraldehyde 3-phosphate dehydrogenase (GAPDH) is shown as an internal control. **c** The amount of pcDNA- nuclear factor kappa-B (NFκB) p50 (0–0.8 μg) was gradually increased and co-transfected with the SNP55C reporter plasmid. **d** The amount of pcDNA- nuclear factor kappa-B (NFκB) p50 (0–0.8 μg) was gradually increased and co-transfected with the SNP55T reporter plasmid

To evaluate the effect of the SNP55 status on *MDM2*-P2 promoter activity, we attempted a luciferase assay. When the Sp1 expression vector and either SNP55T or SNP55C reporter plasmid were co-transfected with COS-1 cells, a slightly higher luciferase activity was detected with SNP55T compared with SNP55C (Fig. 6a). Next, we tested the functional interference between transcription factor Sp1 and NFκB p50 at the SNP55 site. In addition to the first luciferase experiment, we co-transfected a gradually increased amount of the NFκB p50 expression vector (0–0.8 μg; Fig. 6b), and found that activity of *MDM2*-P2 promoter with SNP55C was suppressed in a concentration-dependent manner (Fig. 6c, d; *P* ‹0.05) while that with SNP55T was not (Fig. 6d).

Thus, NFκB p50 appeared to affect *MDM2*-P2 promoter activity, resulting in ASE of *MDM2*.

### Subcellular localization of NFκB p50 in ASE-positive and -negative endometrial cancer tissues

**Fig. 7:**
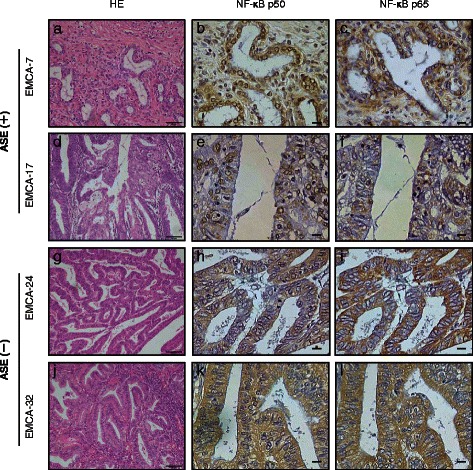
Immunohistochemistry of cancer-free endometrial and endometrial cancer samples. Hematoxylin eosin staining (**a, d, g,** and **j**) and immunohistochemical staining of NFκB p65 (**c, f, i**, and **l**) and NFκB p50 (**b, e, h**, and **k**) in four endometrial cancer samples [Two were allele-specific expression (ASE) positive (+); the others were ASE negative (−)]. Scale bar = 50 μm

NFκB proteins translocate from the cytoplasm to nucleus and exert transcriptional activity. The p50 factor can form a heterodimer with the NFκB/Rel family member p65, and the p50–p65 complex then functions as a transcriptional activator. However, p50 can also form a p50–p50 homodimer, complex that acts as a transcriptional repressor. Therefore, we attempted to confirm the subcellular localization of NFκB p50 and p65 in two ASE-positive and two ASE-negative endometrial cancer specimens by immunohistochemistry. Although we failed to identify a nuclear localization for p50 in ASE-negative endometrial cancer samples (Fig. 7h, k), NFκB p50 localized to the nucleus in ASE-positive samples (Fig. 7b, e); however, p65 did not (Fig. 7c, f). These data indicate that NFκB p50 may have a central role in the ASE of *MDM2* in endometrial cancer.

## Discussion

*MDM2*, one of the most investigated proto-oncogene, plays an important role in tumorigenesis. For a long time, many investigators have attempted to reveal an association between *MDM2*-overexpression, amplification, or genetic variation and increased cancer risk. Ever since the first report by Bond et al. [28], many case–control studies have looked into the roles of SNP309 of *MDM2* in various diseases, particularly cancer, including endometrial cancer. However, the association between endometrial cancer risk and the *MDM2*-SNP309 polymorphism remains controversial.

Our group had previously doubt the association between the status of SNP309 only and endometrial cancer risk [66]. This corresponded to our expectation that the SNP309 status may not affect the transcriptional activity of the *MDM2*-P2 promoter. We also demonstrated that the Ras /ER/MDM2 pathway was critical for NIH3T3 cell line transformation and the blockage of this pathway resulted in an inhibitory effect in estrogen-depended gynecological cancers such as ovarian cancer and endometrial cancer [69–71]. This indicated that *MDM2* is critical to the development of endometrial cancer. Several studies have attempted to demonstrate the molecular mechanism of the promoter activity of *MDM2*-P2 [28, 30, 72]. These studies conducted by *in vitro* experimentation, because of the difficulty in measuring a quantitative difference in RNA transcription between paternal and maternal alleles. Therefore, it remained unclear whether the phenomenon truly existed *in vivo*.

We overcame this problem by utilizing PLACE-SSCP with genomic DNA and RNA prepared from the same clinical samples at the same time. PLACE-SSCP is based on technology developed for precise estimation of SNP-allele frequencies in pooled DNA [67]. We could not detect quantitative differences in transcription beyond 20 %, between the two alleles in all 23 cancer-free endometria analyzed; however, 17 were heterozygous in SNP309.

Two of the other 17 heterozygous samples showed more than 20 % differences in the total mRNA quantity between two alleles in *MDM2*, but none had such transcriptional quantitative differences from the *MDM2-*P1 promoter. Moreover, there were no genetic variations, such as SNPs or microdeletions, to affect the binding affinity of microRNA in the 3’ UTR of ASE-positive samples. These findings suggest that ASE of *MDM2* in endometrial cancer was caused by the *MDM2*-P2 promoter.

We identified a new functional SNP, SNP55, which appeared to be regulated by NFκB. Then we examined SNP55 status in both cancer-free endometria and endometrial cancer samples analyzed by genome DNA sequencing, and found no difference between cancer-free endometria and endometrial cancer samples neither for the genotype frequencies, nor allele frequencies. Thus, we could not show the association of SNP55 polymorphism with endometrial cancers. We then determined the haplotype involving SNP55 and SNP309 in cancer-free endometria (*n* = 45), and assessed linkage disequilibrium. These two SNPs were in moderate-linkage disequilibrium with each other (r^2^ = 0.483).

We expected that both SNP55 status and transcription factor activity may affect on promoter transcriptional activity, and result in allelic expression differences. As expected, two ASE-positive endometrial cancer samples presented nuclear localization of NFκB p50 observed by immunohistochemistry, although two ASE-negative endometrial cancer samples did not. We attempted to find a quantitative difference in MDM2 protein levels, but failed to detect any difference (data not shown). This was probably because the SNP55 status and NFκB p50 activity were dependent only on P2 promoter activity even though MDM2 was produced from mRNAs transcribed from both P1 and P2 promoters.

NFκB is a transcription factor that is important to various physiological processes, particularly inflammation. The NFκB family comprises five members, p50, p52, p65 (Rel A), c-Rel, and RelB, which form homodimers or heterodimers with each other. These proteins share the highly conserved region, the Rel homology domain, which is essential for DNA binding. Only p65, c-Rel, and RelB have a transcriptional activation domain. Because the others lack such a domain, the p50–p50 complex acts as a transcriptional repressor [73]. Although activation of the p65–p50 complex has been intensively investigated, the mechanisms of p50 production, homodimer formation, nuclear translocation, and activation still need to be determined [74]. Hirano et al., showed functional interference between transcription factor Sp1 and the p50 homodimer at the same DNA binding site [68], same as our observation.

## Conclusions

In this study, we identified a new functional SNP on the *MDM2*-P2 promoter, SNP55. We then demonstrated the *MDM2*-P2 promoter was regulated by both transcription factors Sp1 and p50, and that the p50 homodimer suppressed *MDM2*-P2 promoter activity through the transcription factor Sp1/NFκB binding site including SNP55. These findings suggest that both genetic variations and transcription factor activity affect *MDM2* activation in endometrial cancer. Given that *MDM2* appears to play a critical role in endometrial cancer, control of NFκB p50 homodimer provides the possibility for a new therapeutic target.

## Abbreviations

SNP: Single nucleotide polymorphism
ASE: Allele-specific expression
NFκB: Nuclear factor kappa-B
BIA: Biomolecular interaction analysis
PLACE-SSCP: Post-labeling followed by automated capillary electrophoresis under single-strand conformation polymorphism conditions
